# Navigating Technostress in primary schools: a study on teacher experiences, school support, and health

**DOI:** 10.3389/fpsyg.2023.1267767

**Published:** 2023-11-21

**Authors:** Zhuo Wang, Li Zhang, Xinghua Wang, Lei Liu, Cixian Lv

**Affiliations:** ^1^Department of Primary Education, Teacher’s College, Qingdao University, Qingdao, China; ^2^Department of Psychology, Teacher’s College, Qingdao University, Qingdao, China; ^3^Department of Instructional Technology, Teacher’s College, Qingdao University, Qingdao, China; ^4^Center of Educational Research, Teacher’s College, Qingdao University, Qingdao, China

**Keywords:** Technostress, primary teachers, school support, work–family conflicts, personal health

## Abstract

The COVID-19 pandemic has led to a global shift toward online education, which has increased the use of technology for communication, management, and remote teaching. This study aimed to investigate how primary school teachers in China used technology during the Pandemic and to what extent they experienced Technostress, as well as the impact of Technostress on work–family conflicts and technology-induced health issues. A survey was conducted among 1,172 primary school teachers, and the results revealed that teachers exhibited a moderate to a high level of Technostress during the Pandemic, with differences observed in gender, age, and headteacher duties. Furthermore, Technostress was positively correlated with work–family conflicts and technology-induced health issues. Technology use intensity was found to directly impact work–family conflicts and personal health and indirectly impact them via the agency effects of Technostress. School support moderated the indirect relationship between technology use intensity and work–family conflicts and health issues, with higher levels of school support leading to less apparent impacts of technology intensity on work–family conflicts and personal health via the agency effect of Technostress. These findings provide timely insights for post-pandemic teacher training and technology management and suggest the importance of school support in promoting sustainable educational development.

## 1 Introduction

The breakout of COVID-19 has forced many countries to issue strict policies for social distancing and intermittent quarantine (e.g., Shigemura et al., 2020). Worldwide, universities and schools have required their teachers and students to shift from offline classes to partially or fully online classes during severe pandemic times (Patel et al., 2020). For teachers, the abrupt change of teaching mode has naturally incurred more technology use for frequent remote education, communication, and management (e.g., Rey-Merchán and López-Arquillos, 2022).

Indisputably, technology has profoundly changed our ways of life and work, with numerous benefits like increased efficiency and convenience; however, its misuse or overuse may also cause negative effects. This has been manifested by increased phone addiction (Sahu et al., 2019), cyberbullying (Gaffney et al., 2019), and data breaches (Cadwalladr and Graham-Harrison, 2018), among other things. One of the significant issues is “Technostress,” a type of psychological distress caused by technology use (Brod, 1984). Research has concluded that Technostress could impact users’ perceived work overload, cause demotivation, and lead to job dissatisfaction (Ragu-Nathan et al., 2008). However, the majority of Technostress studies have been framed within a business or industrial work context (Jung et al., 2012; Salanova et al., 2014; Tarafdar et al., 2015; Hsiao, 2017; Marchiori et al., 2019), whereas a small but increasing number of studies are now focusing on the educational context (e.g., Joo et al., 2016; Syvänen et al., 2016; Penado-Abilleira et al., 2020).

Teachers, who are increasingly required to integrate emerging technologies into their teaching, management, and communication with students and parents, often struggle with cognitive and psychological technology use (Tarus et al., 2015; Voet and De Wever, 2017). With the breakout and enduring effects of COVID-19 as a backdrop, teachers of all levels of education are encountering even more challenges than ever before (e.g., Mushtaque et al., 2021). According to Wang et al. (2021), primary school teachers who were required to teach online during the quarantine “felt that ‘online teaching and preparation is too exhausting’ and it was hard to ‘maintain such a high level of energy and devotion’ while dealing with their own family affairs at home.” For example, a math teacher who was interviewed reported that she had to make numerous announcements daily via the Dingding platform to keep her students engaged and prevent them from falling behind (Wang et al., 2021).

Additionally, even physical education (PE) teachers, who were least frequently required to use technology before, have been asked to utilize online technologies to create video lectures and distribute exercises for their students (Wang et al., 2021). Given the above, it can be reasonably expected that primary school teachers’ use of technology has increased, leading to increased pressure.

Compared to middle or high school teachers, primary school teachers mainly interact with younger students who are more prone to misbehavior and are more likely to profoundly affect their psychological, cognitive, and behavioral development. For instance, McLean et al. (2023) highlight that elementary teachers, including primary school teachers, are responsible for providing primary, daily instruction in multiple content areas to a single group of students. This suggests that primary school teachers have more direct and consistent interaction with younger students, which can profoundly impact their psychological, cognitive, and behavioral development. Without appropriate policies and interventions, primary school teachers may lack effective strategies to overcome such challenges, jeopardizing their well-being, student outcomes, and overall educational sustainability. Therefore, it is essential to examine the current level of Technostress among primary school teachers, identify potential contributing factors and significant consequences, and provide schools and districts with conducive adaptations that promote teachers’ well-being and, ultimately, student outcomes.

Therefore, the present paper sought to answer the following questions through a survey research design: (1) What is the current status of technology intensity and Technostress among primary school teachers? (2) How do primary school teachers’ technostress levels differ in demographic variables? (3) How do primary school teachers’ technology intensity and perceived school support impact their work–family conflicts? (4) How do primary school teachers’ technology intensity and perceived school support impact their health?

## 2 Literature review

### 2.1 Technostress

Technostress was formally conceptualized and introduced to the public by Brod (1984) in the book Technostress: The Human Cost of the Computer Revolution. Brod defined Technostress as “a modern disease of adaptation caused by the inability to cope with new technologies in a healthy manner” (s.n.). Due to rapid industrialization and modernization, current organizations are characterized by an increasing emphasis on knowledge-intensive work, which requires employees to constantly interact with evolving ICT and make frequent physical, social, and cognitive adjustments. As a result, technology use’s negative effects can lead to physiological and psychological issues. Technostress can cause physiological symptoms such as tiredness (Salanova et al., 2014), crankiness, and insomnia (Porter and Kakabadse, 2006), as well as psychological problems, including a sense of frustration, perceived time pressure, and increased cognitive load (Mark et al., 2008), skepticism, and a sense of incompetence (Salanova et al., 2014). Technostress can also indirectly affect organizational outcomes, such as deteriorated work ethics, dissatisfaction with work, work-life conflicts, and reduced employee productivity (Tarafdar et al., 2007).

In addition to examining the effects of Technostress, previous literature also sought to conceptualize and measure Technostress. As was mentioned earlier, Technostress refers to stress experienced by end users resulting from their usage of information and communication technologies. Essentially, it is a type of stress and may be understood from the organizational behavior literature. Based on the Transaction-Based Model, stress is deemed as a combination of stressors (i.e., the events, demands, stimuli or conditions that individuals encounter and appraise as potentially exceeding their capabilities) and the individuals’ cognitive, emotional, and behavioral responses to those stressors. Therefore, Ragu-Nathan et al. (2008) conceptualized Technostress as stemming from factors that create stress from use of ICT and end-users’ response to such stress (e.g., feeling worried or sacrificing leisure time for work). By referring to both extant literature and practitioner observations, Ragu-Nathan et al. (2008) identified five dimensions of Technostress creators; namely, they were techno-invasion, techno-overload, techno-complexity, techno-insecurity, and techno-uncertainty. To assess these dimensions, they developed a scale with questions corresponding to each one. Although the scale was originally developed to measure responses from 680 white-collared organizational end users, the questions asked are sufficiently general to also apply to other groups that regularly use ICT such as teachers, small business owners, and teleworkers. For example, Califf et al. (2020) adapted this scale to measure 204 nurses’ Technostress, while Jena (2015) surveyed 216 academicians in India using an adapted version as well. Overall, Ragu-Nathan et al. (2008) scale provides a useful, validated instrument for assessing multiple facets of Technostress experienced by users of modern ICT across a variety of contexts. Specifically, techno-invasion is defined as the highly diffused use of technologies without any limitation or consideration of space and time, putting users at a high risk of being interrupted out of business hours (Tarafdar et al., 2007; Gaudioso et al., 2017). Techno-overload occurs when an employee receives the same information from multiple channels simultaneously, causing cognitive redundancy and repetition (Tarafdar et al., 2007; Gaudioso et al., 2017). Techno-complexity is a negative feeling that the target ICT is difficult to learn and can take tremendous effort to master. Techno-insecurity is depicted as the perception that ICT are frequently updated, which could eventually replace human beings’ jobs (Tarafdar et al., 2007). Finally, techno-uncertainty denotes the situation when a user perceives the introduction of new ICT as a manifestation of instability and unpredictability (Tarafdar et al., 2007).

The third strand of research focuses on the causes or contributing factors of Technostress, which have been suggested to include lack of institutional support (Wang et al., 2008; Salanova et al., 2014), fatigue (Khuntia et al., 2015), interruptions resulting from multitasking (Mark et al., 2008; Ragu-Nathan et al., 2008; Tarafdar et al., 2015), absence of effective personal coping mechanisms (Rohwer et al., 2022), and other demographic factors (Ursavaş et al., 2011; Tarafdar et al., 2015).

It has also been pointed out that the level of Technostress seems to vary by gender and age. For example, some findings indicate that older employees might not experience as much Technostress as their younger counterparts (e.g., Ragu-Nathan et al., 2008), probably because the former had already become accustomed to the work environment and thus could handle related stress more effectively than the latter (Tarafdar et al., 2015). As for gender differences in Technostress, mixed results have been reported. For instance, Ong and Lai (2006) found that women generally experienced more computer anxiety than men, while Ragu-Nathan et al. (2008) concluded that male employees suffered from Technostress more, and some other studies found no gender difference at all (Shah et al., 2012).

### 2.2 Teachers’ Technostress

“The complexity and intensity of the pressures on teachers and the pace of education reform are unprecedented” (Grenville-Cleave and Boniwell, 2012, p. 3). Golembiewski et al. (1983) deduced that the teaching profession strongly correlated with elevated work stress. Uzair and Bhaumik (2023) also emphasized that teaching ranks among the most stressful professions globally due to continuous changes in scientific and technological advances from the 1990s to the present. Teachers’ roles are expected to evolve from mere “transmitters of knowledge” to instructional designers possessing expertise in pedagogy, technology, and content knowledge. Compared to other professions, teachers are likely among the first to benefit from and suffer due to technological advancements. On the one hand, teaching as a profession demands work beyond classroom time or regular working hours, which associates with high levels of burnout (Ernst Kossek and Ozeki, 1998; Hakanen et al., 2006). Such distinctive characteristics of the teaching profession could potentially induce considerable stress and psychosocial problems (Dussault and Deaudelin, 1999; Fullan, 2001).

On the other hand, given the strategic role of education in any country, teachers are often expected to master innovative technologies to revolutionize teaching and nurture competitive global citizens for the future. For example, driven by educational initiatives like TPACK, teachers must now devote greater attention to integrating technology into teaching (Graham, 2011). Ultimately, it is recognized that teachers’ ability to integrate technology into students’ meaningful learning is crucial to the success of educational innovation (Schildkamp et al., 2020).

Despite this, teachers often need help with the timely and efficient acquisition of relevant skills due to the constant emergence and updating of new technologies (Tarus et al., 2015). Teachers either lack access to such knowledge (Altınay-Gazi and Altınay-Aksal, 2017; Li and Wang, 2021) or when they do have access, their limited time precludes frequent technology learning—in a comparative study between teachers (*n* = 150) and non-teachers (*n* = 148), Grenville-Cleave and Boniwell (2012) discovered that teachers’ perceived well-being was statistically significantly lower than that of non-teachers. A national survey of 24,100 teachers from 428 schools in Britain suggested that teachers’ overall well-being strongly correlated with students’ performance (Briner and Dewberry, 2007, as cited in Atteh et al., 2020, p. 50). Given the prolonged daily interaction between primary school teachers and students, ensuring teachers’ mental and physical health is essential.

Nonetheless, existing research on teachers’ Technostress remains limited. An advanced search with Technostress and teachers as abstract keywords yielded only 15 full-text papers. Among these, nine included teachers of mixed educational levels (e.g., Hassan et al., 2018; Dong et al., 2020; Khlaif et al., 2022), four focused on university teachers (e.g., Li and Wang, 2021), one on high school only (Magistra et al., 2021), and one on secondary school only (Joo et al., 2016). While these studies have provided insightful contributions to Technostress’ creators, inhibitors, and mediators, none exclusively focused on primary teachers. Moreover, the role of significant variables, such as work–family conflicts and teachers’ well-being, has yet to be examined in these studies. As is pointed out by Cerrato and Cifre (2018), there is growing interest in investigating work–family conflicts in organizational psychology research concerning job performance and satisfaction. Previous research indicates that employees’ deep involvement in home affairs impedes desirable job participation and productivity (Huang et al., 2004). Some known factors impacting work-life conflict include excessive workload, ambiguous roles, organizational culture, and work environment (Atteh et al., 2020). It has been argued that “teachers experiencing high rates of work–family conflicts end up with extended periods of stress, become disconnected from their duties and tasks, and have poor job satisfaction” (Ernst Kossek and Ozeki, 1998, as cited in Atteh et al., 2020, p. 50). This is particularly true for headteachers, who are expected to fulfill more diverse roles than non-headteachers, such as establishing cultural identification, managing resources, and maintaining good relationships with parents (Argyriou and Iordanidis, 2014). For instance, a study conducted on 359 headteachers revealed that two-thirds of them reported experiencing high levels of work stress (Scott et al., 2021). Additionally, the teaching profession is frequently associated with certain physical ailments, such as rhinopharyngitis/laryngitis, conjunctivitis, bronchitis, eczema/dermatitis, and varicose veins (Kovess-Masféty et al., 2006). Examining teachers’ work–family conflicts and health conditions is equally important because teachers’ well-being is positively correlated with students’ well-being (Harding et al., 2019).

Therefore, a comprehensive picture of the variables above would likely contribute to a more systematic and holistic understanding of Technostress. Policymakers and school administrators can utilize such information to assist teachers with varying levels of Technostress and even prevent the increase of Technostress with appropriate measures. By addressing work–family conflicts and teachers’ well-being, stakeholders can enhance teachers’ overall satisfaction and performance, ultimately benefiting students’ learning outcomes and the quality of education.

## 3 Theoretical framework

This study is based on the Conservation of Resources (COR) theory, which provides a comprehensive framework for understanding how individuals react to stress and its impact on their well-being. According to the COR theory (Hobfoll, 1989), individuals strive to acquire, retain, and protect resources they value, like time, energy, skills, and psychological well-being. Stress occurs when there is a threat to or loss of these resources, leading to adverse outcomes. Zhang et al. (2022) provide further evidence by discussing the impact of traumatic events, such as natural disasters, on resource loss. The study identifies four types of resource loss: objects, conditions, personal characteristics, and energies. The threat of resource loss, actual resource loss, or lack of resource gain can lead to psychological stress. In the context of this study, the resources of interest include time, energy, skills, and psychological well-being.

Primary school teachers face challenges posed by increased technology use during the COVID-19 pandemic (Wang et al., 2021). Communication, management, and remote teaching technology have become essential in the transition to online education. However, this increased reliance on technology may result in Technostress, which refers to the stress and strain experienced when individuals perceive technology demands as exceeding their available resources.

By adopting the COR theory, this study examines the impact of Technostress on primary school teachers’ work–family conflicts and technology-induced health issues. Furthermore, it explores the role of technology use intensity and school support as important factors that may influence the relationships between Technostress and these outcomes.

Drawing on the COR theory, this study generates hypotheses regarding the relationships between Technostress, technology use intensity, work–family conflicts, personal health, and the moderating role of school support. By integrating this theoretical framework into our analysis, we aim to provide a comprehensive understanding of the mechanisms underlying the experiences of primary school teachers navigating Technostress during the Pandemic.

*Hypothesis* 1: Primary school teachers’ level of technology intensity and Technostress will be high due to the impact of the Pandemic, which disrupted their usual resources and teaching activities.

This hypothesis aligns with the COR theory, which suggests individuals strive to acquire, retain, and protect resources. The pandemic-induced disruptions have likely led to a depletion of resources, such as access to technological tools, training, and support, resulting in increased Technostress among primary school teachers. Previous studies have examined the effects of the COVID-19 pandemic on education and technology use in the teaching profession (Rasmitadila et al., 2020). These studies provide valuable insights into the relationship between the Pandemic, technology intensity, and Technostress among primary school teachers.

*Hypothesis* 2: Primary school teachers’ Technostress levels will vary based on demographic variables. Specifically, female and older teachers, as well as headteachers may experience higher levels of technostress than their counterparts.

This hypothesis aligns with the COR theory as it acknowledges that demographic factors, such as age and gender, can influence the allocation and availability of resources. The theory posits that individuals may experience resource depletion or strain based on these demographic characteristics, which can contribute to differences in technostress levels. Research findings suggest age and gender differences in technostress experiences, although the results are mixed. Older employees tend to experience less Technostress than their younger counterparts (Ragu-Nathan et al., 2008), and women experience more computer anxiety than men (Ong and Lai, 2006). However, some studies find no significant gender difference in technostress levels (Shah et al., 2012).

*Hypothesis* 3: Higher levels of technology intensity among primary school teachers will be associated with increased work–family conflicts moderated by perceived school support.

This hypothesis aligns with the COR theory by recognizing that higher technology intensity can deplete resources and increase work–family conflicts. The moderating effect of perceived school support suggests that the availability of resources (support) can buffer the negative impact of technology intensity on work–family conflicts, aligning with the protective and buffering role of resources proposed by the COR theory. Technostress has been associated with various factors, including lack of institutional support (Wang et al., 2008; Salanova et al., 2014), interruptions caused by multitasking (Mark et al., 2008; Ragu-Nathan et al., 2008; Tarafdar et al., 2015), and inadequate personal coping mechanisms (Rohwer et al., 2022).

*Hypothesis* 4: Higher levels of technology intensity among primary school teachers will be associated with poorer personal health outcomes, moderated by perceived school support.

This hypothesis aligns with the COR theory by suggesting that higher technology intensity can deplete resources, leading to poorer personal health outcomes. The moderating effect of perceived school support implies that the availability of resources (support) can mitigate the negative impact of technology intensity on personal health, aligning with the protective and buffering role of resources proposed by the COR theory. For example, Aktan and Toraman (2022) highlighted the negative impact of long-term screen use on teachers’ health, including various health problems and technology addiction. The sudden shift to online teaching during the Pandemic has also contributed to psychological and emotional problems for teachers, resulting in burnout and other mental health issues (Stockwell and Wang, 2023).

## 4 Methods

### 4.1 Research design

Through a survey research methodology, this study employed a comprehensive questionnaire to gather pertinent data. The survey was accessible from October 8th to November 28th, 2021, collecting 1,172 valid and anonymous responses. Data acquisition was facilitated through WJX.cn, a prominent online questionnaire platform extensively utilized in China for research purposes. This approach allowed for the efficient and secure collection of valuable insights, which were subsequently analyzed to further our understanding of the subject matter.

### 4.2 Participants

The study’s participants comprised primary teachers from two distinct provinces and districts in eastern China. The sample was predominantly female, with 941 (80.3%) female teachers and 231 (19.7%) male teachers. Most respondents (*n* = 496, 42.2%) fell within the age bracket of 31 to 40. Regarding teaching experience, a substantial proportion (45.5%) of the participants were seasoned teachers with over a decade of experience, while 28.1% were relatively new to the profession, having accrued less than 3 years of experience. This diverse sample allowed for a robust exploration of perspectives across various demographic and professional backgrounds.

### 4.3 Ethical approval

This study received ethical approval from the Ethical Committee Review Board at Teacher’s College of Qingdao University. The research procedures, including data collection and participant anonymity, were ensured in strict accordance with the ethical guidelines and standards established by our institution. Participants’ informed consent was obtained upon clicking corresponding buttons in the online platform, and every effort was made to protect their confidentiality and privacy throughout the study.

### 4.4 Data collection

The lead investigators initiated the data collection by disseminating the online survey link to a reputable teacher-researcher within the target region. This individual subsequently extended invitations to 14 primary school principals, who voluntarily assisted in distributing the survey link to a broader network of teachers. Informed consent was secured as potential participants were presented with the study description and voluntarily agreed to partake by selecting the appropriate buttons on the survey webpage. All completed surveys were submitted anonymously to ensure confidentiality and promote candid responses.

### 4.5 Instruments

The comprehensive questionnaire encompassed 45 items, organized into six distinct sections: demographic information (10), technology intensity (5), Technostress (15), school support (5), work–family conflicts (5), and personal health issues (5). The demographic section comprised 10 questions to elicit relevant background information, including participants’ gender, age, and years of teaching experience, among other pertinent details. This structured approach facilitated the systematic collection and analysis of data to understand better the multifaceted relationships between the various factors under investigation.

The technology intensity dimension aimed to assess the daily duration primary school teachers devoted to digital technology utilization across various professional responsibilities, including teaching, lesson preparation, administrative tasks, communication with students’ parents, and attending to personal matters. This section featured five custom-designed questions: “How long do you spend preparing lessons with technologies daily?” The internal consistency of this dimension, as indicated by Cronbach’s α, was determined to be 0.66, suggesting an acceptable level of reliability. It should be cautioned that we deliberately decided to utilize different reply options for a long time in designing these questions. This decision was based on the nature of the activities and tasks associated with technology use among the participants. Some technology-intensive tasks (e.g., lesson preparation and completing administrative tasks) required a more specific time range, ranging from 30 min to 2 h, to capture those activities’ duration accurately.

On the other hand, other technology-intensive tasks were better represented using a broader time frame, including reply options of 1, 2, 3, and 4 h (i.e., the time spent on teaching with technology). By providing a range of response options, we aimed to ensure that participants could select the option that best reflected their engagement in technology-related activities. This approach allowed for a more nuanced and accurate assessment of technology intensity among the participants. We acknowledge that using different reply options may introduce some variability in the data, but we believe that the benefits of capturing a comprehensive picture of technology intensity outweigh the potential limitations introduced by the varied response options.

Adapted from Ragu-Nathan et al. (2008) work, which consisted of 28 questions on Technostress creators (i.e., techno-overload, techno-uncertainty, techno-complexity, techno-invasion, and techno-insecurity), we tentatively eliminated those that were assessed as inappropriate or unnecessary for capturing primary school teachers’ actuality in China upon rigorous discussion. Afterwards, 15 questions were initially kept to be used concertedly with other survey constructs. Confirmatory factor analysis (CFA) of the five-dimensional Technostress scale revealed that items techno-overload 1 (0.49) and techno-invasion 3 (0.34) exhibited underestimation of the corresponding dimensions. Upon removal of these two items, the model demonstrated a satisfactory fit with *χ^2^*/pdf = 4.67, CFI = 0.95, TLI = 0.93, RMSEA = 0.08, and SRMR = 0.05, thus indicating strong construct validity of the scale. The overall Cronbach’s α for all items in the current study was 0.89, while Cronbach’s α values for each dimension were 0.79, 0.81, 0.89, 0.65, and 0.84. An example statement from this section is, “Due to the pervasive use of technology, your time spent with your family has decreased.” The response scale ranged from 1 (Strongly disagree) to 5 (Strongly agree).

The school support scale featured five items adapted from Lam et al. (2010) work, with respondents being asked to indicate their level of agreement with statements such as, “The training provided by our school helped me understand how to integrate technology into teaching.” The scale demonstrated high reliability, with a Cronbach’s α of 0.91.

The work–family conflicts scale included five questions from Carlson et al.'s (2000) research, with a Cronbach’s α of 0.89. Participants were prompted to indicate their agreement level with the statements, utilizing a range of 1 (Strongly disagree) to 5 (Strongly agree). Higher scores signified increased work–family conflicts.

Lastly, personal health issues were assessed through five custom-designed items, inquiring about the frequency of technology-related health concerns, such as “How frequently do you feel visual tiredness because of technology use?” The scale exhibited strong reliability, with a Cronbach’s α of 0.93. A higher score denoted more frequent experiences of technology-induced health symptoms.

### 4.6 Data analysis

SPSS26.0 was used to perform descriptive statistics, bivariate analysis, and difference tests. Moderated mediation effects were tested by PROCESS v3.4.0.

## 5 Results

### 5.1 Primary teachers’ technology intensity and Technostress during the pandemic

**Figure 1:**
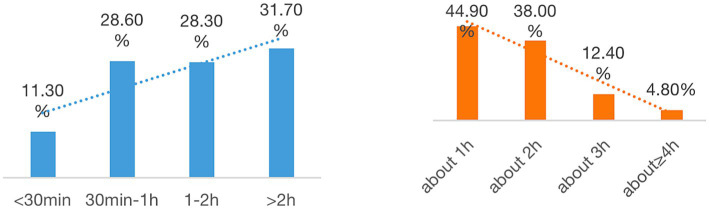
Daily time spent on lesson planning (left) and teaching (right).

Figure 1 illustrates the distribution of time primary school teachers spend in China on lesson preparation using digital technology. A notable 31.7% of respondents reported dedicating more than 2 h to this task, while 28.3% allocated between one and 2 h. A smaller percentage (11.3%) indicated they spent less than 30 min utilizing technology for lesson preparation.

Concerning technology use in teaching, most primary school teachers (82.9%) devoted approximately one to 2 h daily to this aspect of their practice. A small proportion of teachers (4.8%) reported an exceptional 4 h or more of daily technology use in teaching. This data highlights the variability in technology adoption and utilization across primary school classrooms in China.

**Figure 2:**
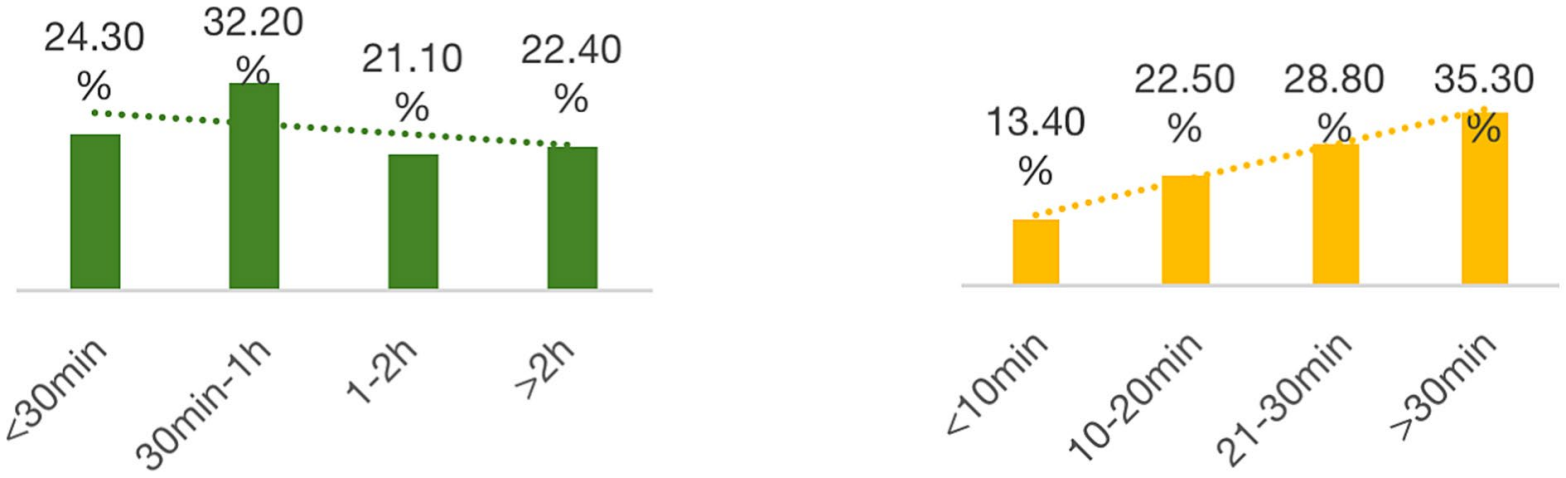
Daily time on administrative tasks (left) and parent communication (right).

Furthermore, Figure 2 (left) presents a relatively even distribution of teachers’ time spent on administrative tasks using digital technology. A majority (56.5%) reported spending less than 1 h, while 21.1% allocated between one and 2 h, and 22.4% devoted more than 2 h to these tasks. Regarding communication with students’ parents via technology (Figure 2, right), the largest proportion of teachers (35.3%) indicated spending over 30 min on this activity. This finding highlights the diverse range of time commitments associated with various aspects of technology use in the professional lives of primary school teachers.

**Figure 3:**
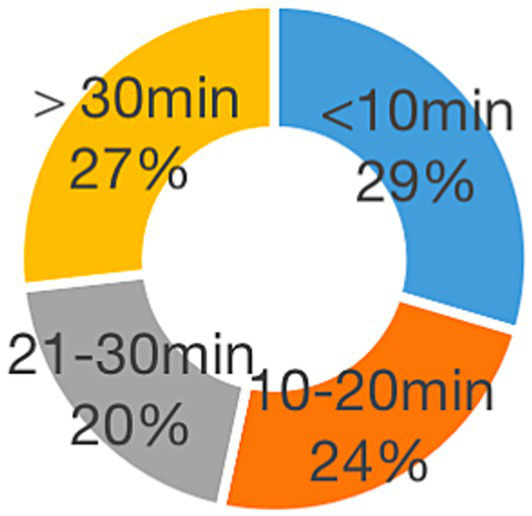
Daily time spent resolving personal affairs with technology.

Regarding time spent using technology for personal affairs (Figure 3), most teachers (73%) reported devoting less than 30 min daily, while 27% indicated spending over 30 min on such activities. Considering all aspects of technology use, it can be inferred that primary school teachers engage with digital tools for approximately 4 to 6 h per day, irrespective of the specific purposes for which they are employed. This highlights the pervasive role of technology in teachers’ professional and personal lives.

Regarding the Technostress level (refer to Table 1), the data reveals that primary school teachers, on average, experience a moderate to high level of Technostress, with a mean score of 3.41 on a scale of 1 to 5. In other words, Hypothesis 1 was supported. Specifically, among the five sub-categories, techno-uncertainty and techno-overload scores were notably higher than those of the other dimensions. At the same time, techno-complexity registered the lowest score—a repeated measures ANOVA was subsequently conducted to investigate differences between dimensions.

**Table 1:** Descriptive statistics of primary school teachers’ technostress (*N* = 1,172).

Rank	Dimension	N	*M*	*SD*
	Technostress	1,172	3.41	0.81
1	*Techno-uncertainty*	1,172	3.78	1.02
2	*Techno-overload*	1,172	3.55	1.07
2	*Techno-invasion*	1,172	3.51	1.21
3	*Techno-insecurity*	1,172	3.27	0.92
4	*Techno-complexity*	1,172	3.12	1.14

The higher the ranking, the more technostress the teachers perceived.

Before conducting the repeated measures ANOVA, we initially assessed the normality of the data using the Kolmogorov–Smirnov (K-S) and Shapiro–Wilk (S-W) tests on the five dimensions of Technostress. The results of these tests indicated that all five dimensions had *p* < 0.001, suggesting a potential violation of the normality assumption. However, it is important to note that upon further examination, including the review of histograms and normal curves of the comprehensive dataset, we observed that the absolute values of kurtosis and skewness for the five dimensions of Technostress were all less than 1. This observation aligns with the criterion suggested by Gravetter and Wallnau (2014) and Field (2000), where absolute values less than 2 are considered indicative of data that approximates a normal distribution. Additionally, considering our large sample size (*N* = 1,172), it is reasonable to approximate that the data follows a normal distribution, making parametric tests appropriate for our analysis. We chose to employ parametric tests due to their robustness when the data is normally distributed. We acknowledge that the nonparametric tests yielded consistent results with the parametric tests, but we opted for parametric analyses to maximize the sensitivity of our statistical tests and adhere to established practices in the field.

To provide a more accurate description of the data results, the present study further employed the Friedman test, which revealed significant differences in the five dimensions of Technostress among the teachers (*Z* = 396.94, *p* < 0.001). Specifically, teachers perceived the highest levels of techno-uncertainty, followed by techno-overload and techno-invasion (with no significant difference observed between the latter two dimensions, *Z* = 0.385, *p* = 1), and the lowest levels of techno-insecurity and techno-complexity. The results from the nonparametric test were consistent with those obtained from the parametric test. Following the recommendations of Gravetter and Wallnau (2014) and Field (2000), the data in this study were considered to approximate a normal distribution, and the results of the parametric tests were used.

To sum up, results of Friedman rank sum tests indicated that there were statistically significant differences among the five Technostress dimensions overall (*p* < 0.05). Follow-up comparisons showed that the means for techno-overload and techno-invasion were not significantly different from each other, but were significantly higher than the means for techno-insecurity, techno-complexity, and techno-uncertainty (*p* < 0.05). Additionally, pairwise comparisons revealed that the means for techno-insecurity, techno-complexity, and techno-uncertainty did not differ statistically from each other. To summarize, the Friedman test revealed significant differences among groups overall, while post-hoc analyses showed techno-overload and techno-invasion were highest and equivalent, and techno-insecurity, complexity, and uncertainty were lower but equivalent.

### 5.2 Demographic differences in primary teachers’ Technostress

In the above section, we demonstrated that the data on Technostress among primary school teachers approximates a normal distribution, including the overall mean score of Technostress. Therefore, a one-way ANOVA examined significant differences between age and teaching experience. At the same time, t-tests were used to assess gender and headteacher (yes/no) differences in Technostress.

Kruskal-Wallis tests were performed to provide a more accurate description of the data results to analyze differences between different age and teaching experience groups. The results revealed significant differences in Technostress among teachers based on age (*H* = 90.25, *p* < 0.001), indicating that higher age was associated with higher perceived Technostress. Additionally, there were significant differences in Technostress based on teaching experience (*H* = 119.67, *p* < 0.001), indicating that longer teaching experience was associated with higher perceived Technostress. Using the U-Mann–Whitney test, significant gender differences were observed in Technostress (*Z* = 4.16, *p* < 0.001), with male teachers reporting significantly higher levels of Technostress than female teachers. Furthermore, headteachers (yes/no) also exhibited significant differences in Technostress (*Z* = 2.27, *p* < 0.05), with headteachers reporting significantly higher levels of Technostress compared to non-headteachers. The results from the nonparametric tests were consistent with those obtained from the parametric tests. Following the recommendations of Gravetter and Wallnau (2014) and Field (2000), the data in this study were considered to approximate a normal distribution, and the results of the parametric tests were used. Therefore, Hypothesis 2 was supported as well (Table 2).

**Table 2:** Demographic differences in primary teachers’ technostress.

Demographics	Distribution	Technostress		
		M ± SD	*F/t*	*p*
Age	20–30 (*n* = 330, 28.2%)	3.12 ± 0.83	29.59	<0.001***
	31–40 (*n* = 494, 42.2%)	3.42 ± 0.78		
	41–50 (*n* = 260, 22.2%)	3.65 ± 0.75		
	51–60 (*n* = 88, 7.5%)	3.76 ± 0.67		
Gender	Male (*n* = 231, 80.3%)	3.62 ± 0.85	4.30	<0.001***
	Female (*n* = 941, 19.7%)	3.36 ± 0.79		
Teaching years	< 3 years (*n* = 329, 28.1%)	3.10 ± 0.78	39.04	<0.001***
	3–5 years (*n* = 151, 12.9%)	3.24 ± 0.81		
	5–10 years (*n* = 159, 13.6%)	3.39 ± 0.79		
	>10 years (*n* = 533, 45.5%)	3.66 ± 0.75		
Headteacher or not	Headteacher (*n* = 628, 53.6%)	3.46 ± 0.82	2.22	0.026*
	Non-headteacher (*n* = 544, 46.4%)	3.36 ± 0.79		

* *p* < 0.05; ** *p* < 0.01; *** *p* < 0.001.

### 5.3 Impact of primary teachers’ technology use and perceived school support on their work–family conflicts

Given that all variables were collected through self-reported questionnaires, assessing the potential for common method bias was necessary. Harman’s single-factor test (Podsakoff et al., 2003) revealed nine factors with eigenvalues greater than 1. The largest unrotated common factor accounted for 27.29% of the variance, which falls below the 40% critical threshold (Tang and Wen, 2020). These findings suggest that common method bias was not a significant concern in the present study.

The mean, standard deviation, and correlation matrix for each variable are presented in Table 3. Correlation analysis revealed that Technostress was significantly positively correlated with technology intensity, school support, work–family conflicts, and personal health issues. Conversely, school support demonstrated a significant negative correlation with Technostress, technology intensity, work–family conflicts, and health issues. Age positively correlated with Technostress, work–family conflicts, and personal health issues while negatively correlated with technology intensity.

**Table 3:** Descriptive statistics and correlations among variables of interest.

	1	2	3	4	5	6	7	8
Gender	—							
Age	N/A	—						
Headteacher	−0.09^**^	−0.15^**^	—					
Technology intensity	−0.16^**^	−0.12^**^	0.25^**^	—				
Technostress	0.13^**^	0.28^**^	0.07^*^	0.09^**^	—			
W-F conflicts	0.06	0.12^**^	0.08^**^	0.13^**^	0.45^**^	—		
Health impact	0.07^*^	0.17^**^	0.11^**^	0.15^**^	0.55^**^	0.68^**^	—	
School support	0.03	−0.02	−0.02	−0.07^*^	−0.06^*^	−0.17^**^	−0.18^**^	—
*M*	—	—	—	2.46	3.41	3.45	3.39	3.88
*SD*	—	—	—	0.67	0.81	1.03	1.06	0.82

* *p* < 0.05; ** *p* < 0.01; W-F refers to work-family.

Regarding research question 2, results indicated gender differences in primary teachers’ Technostress (*t* = 4.30, *p* < 0.001). Additionally, work–family conflicts may arise from excessive workload, and previous research has found that primary school headteachers experience higher levels of job dissatisfaction and stress than their colleagues (Chaplain, 2001). Consequently, gender, age, and headteacher status were incorporated into the current study as control variables (Table 3).

#### 5.3.1 The mediating effect of Technostress

As presented in Table 4, technology intensity significantly and positively predicted Technostress (*β* = 0.14, *p* < 0.001) and work–family conflicts (*β* = 0.13, *p* < 0.01). Additionally, Technostress demonstrated a significant positive predictive effect on work–family conflicts (*β* = 0.54, *p* < 0.001), even after controlling for teachers’ gender, age, and headteacher status. These findings suggest that Technostress partially mediates the relationship between technology intensity and work–family conflicts.

**Table 4:** Model testing of the effects of technology intensity on work–family conflicts.

Predictors	Model 1 (Technostress)		Model 2 (work–family conflicts)	
	*β*	*t*	*β*	*t*
Gender	0.15	2.50**	0.07	0.91
Age	0.24	9.13***	0.004	0.15
Headteacher	0.14	2.99**	0.07	1.32
Technology intensity	0.14	4.09***	0.13	3.03**
School support	−0.04	1.59	−0.19	−5.79***
Technology intensity×School support	−0.08	−2.12*	0.07	1.43
Technostress×School support			0.06	1.76
Technostress			0.54	14.97***
*R* ^2^	0.10		0.24	
*F*	22.30***		45.44***	

* *p* < 0.05; ** *p* < 0.01; *** *p* < 0.001.

To further assess the magnitude of the indirect effect, 5,000 bootstrapping samples were generated from the original dataset using random sampling. The results indicated an indirect effect of 0.08, with a standard error (SE) of 0.02 and a 95% confidence interval (CI) of [0.04, 0.12]. As the empirical 95% CI does not include zero, it can be concluded that technology intensity significantly indirectly affects teachers’ work–family conflicts.

#### 5.3.2 Testing the moderated mediating effect of school support

The results of multiple linear regression revealed that technology intensity significantly and positively predicted Technostress (*β* = 0.14, *p* < 0.001), while the interaction between technology intensity and school support (*β* = −0.08, *p* < 0.05) significantly and negatively predicted Technostress (Table 4, Model 1), after controlling for covariates. The simple slope test results demonstrated that when the level of school support was low (M-1SD), technology intensity significantly and positively predicted teachers’ Technostress (*β* = 0.21, *t* = 4.39, *p* < 0.001). Conversely, when the level of school support was high (M + 1SD), technology intensity had no significant predictive effect on Technostress (*β* = 0.05, *t* = 0.93, *p* = 0.35).

**Figure 4:**
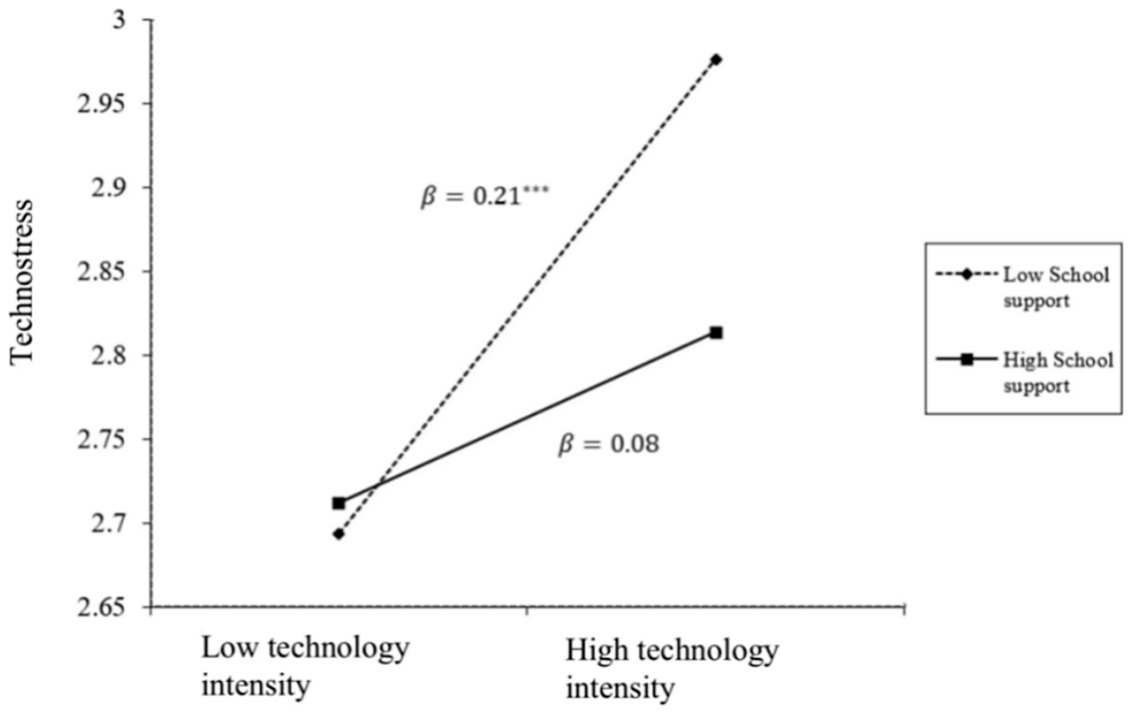
The moderating effect of school support.

These findings indicate that school support serves as a moderator in the relationship between technology intensity and Technostress. For teachers perceiving low school support, technology intensity was a significant positive predictor of their Technostress. However, the predictive effect was insignificant for those who perceived high school support. The moderating effect is illustrated in Figure 4.

**Figure 5:**
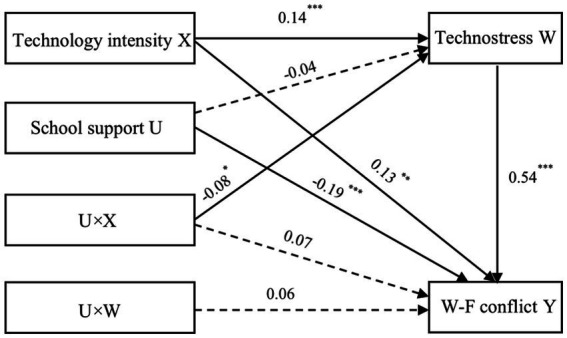
The moderated mediation model of the relationship between technology intensity and teachers’ work–family conflicts.

As shown in Table 4 (Model 2), the interaction between school support and technology intensity did not predict work–family conflicts (β = 0.07, *p* = 0.15), and so did the interaction between school support and Technostress (*β* = 0.06, *p* = 0.08). These suggested that school support only moderated the first stage of the mediation process, not the direct and second stages (Figure 5).

Table 5 presents the moderating effect of school support on the direct and indirect effects. When school support was low (M-1SD), with the 95% CI = [0.06, 0.15] excluding zero, technology intensity significantly and positively predicted work–family conflicts through Technostress. However, the indirect effect was insignificant when high school support (M + 1SD), with the 95% CI = [−0.05, 0.25] containing zero. As school support improved, the direct effect increased while the indirect effect decreased. These results suggest that, with the increase in school support levels, technology intensity is more likely to directly and positively predict teachers’ work–family conflicts. Therefore, Hypothesis 3 was supported.

**Table 5:** Decomposition of direct and mediating effects.

Work–family conflicts	School support	Effect size	Boot SE	Boot LLCI	Boot ULCI	Ratio
Direct effect	−0.88(M – 1SD)	0.07	0.06	0.25	−0.05	39.72%
	0.12(M)	0.13	0.04	0.001	0.05	64.89%
	1.12(M + 1SD)	0.20	0.07	0.002	0.07	86.51%
The indirect effect of Technostress	−0.88(M – 1SD)	0.10	0.02	0.06	0.15	60.28%
	0.12(M)	0.07	0.02	0.03	0.11	35.11%
	1.12(M + 1SD)	0.03	0.04	−0.05	0.12	13.49%

### 5.4 Impact of primary teachers’ technology intensity and perceived school support on their health issues

#### 5.4.1 The mediating effect of Technostress

As shown in Table 6, technology intensity significantly positively predicted Technostress (*β* = 0.14, *p* < 0.001) and health issues (*β* = 0.15, *p* < 0.001), while Technostress had a significant positive prediction effect on health issues (*β* = 0.67, *p* < 0.001), even after controlling for teacher’s gender, age and headteacher or not. These indicated that Technostress partially mediated the association between technology intensity and health issues. We further generated 5,000 bootstrapping samples from the original dataset by random sampling to assess the size of the indirect effect. The results showed that the indirect effect was 0.09, SE = 0.03, 95%CI = [0.04, 0.14]. Empirical 95% CI did not consist of zero, indicating that technology intensity significantly indirectly affected teachers’ health issues.

**Table 6:** Model testing of the effects of technology intensity on health issues.

Predictors	Model 3 (Technostress)		Model 4 (Health issues)	
	*β*	t	*β*	t
Gender	0.15	2.50^**^	0.04	0.63
Age	0.24	9.13^***^	0.05	1.62
Headteacher	0.14	2.99^**^	0.11	2.09^*^
Technology intensity	0.14	4.09^***^	0.15	3.65^***^
School support	−0.04	1.59	−0.18	−5.89^***^
Technology intensity×School support	−0.08	−2.12^*^	−0.004	−0.10
Technostress×School support			0.07	1.87
Technostress			0.67	19.39^***^
*R* ^2^	0.10		0.34	
*F*	22.30^***^		74.53^***^	

* *p* < 0.05; ** *p* < 0.01; *** *p* < 0.001.

#### 5.4.2 Testing the moderated mediating effect of school support

**Figure 6:**
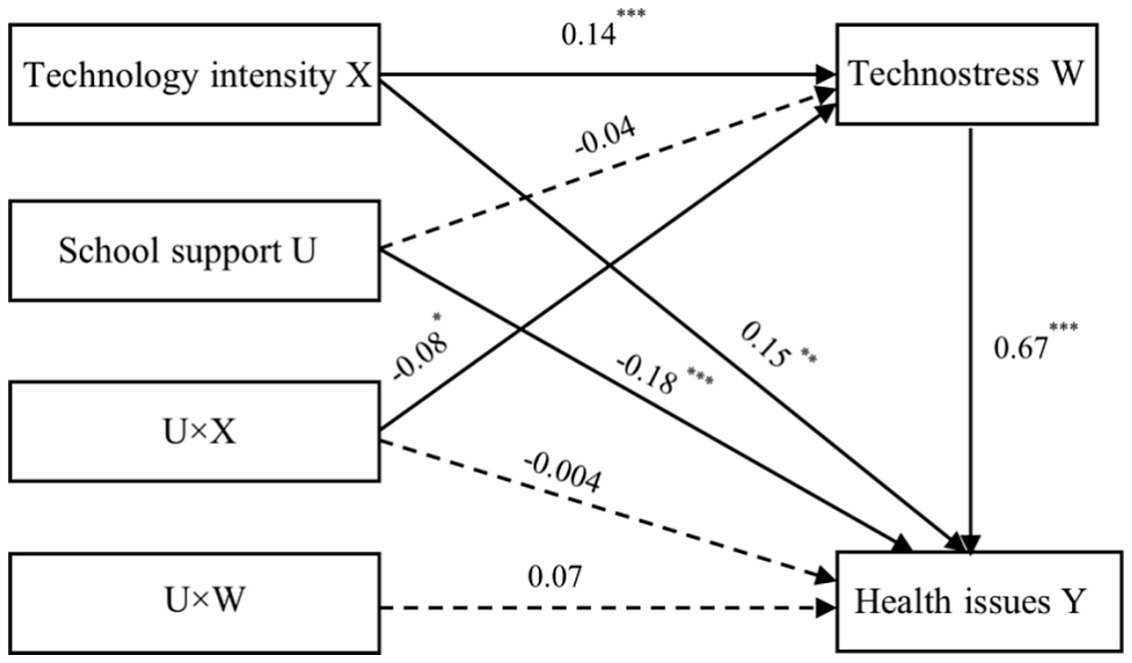
The moderated mediation model of the relationship between technology intensity and teachers’ health issues.

As shown in Table 6 (Model 3) and Figure 4, school support moderated the relationship between technology intensity and Technostress. Table 6 (Model 3) showed that the interaction between school support and technology intensity did not predict health issues (*β* = −0.004, *p* = 0.92), and so did the interaction between school support and Technostress (*β* = 0.07, *p* = 0.06). These suggested that school support only moderated the first stage of the mediation process, not the direct and the second stage. We plotted the moderated mediation in Figure 6.

Table 7 shows the moderating effect of school support on the direct and indirect effects. When school support was low (M-1SD), the 95% CI = [0.09, 0.19] excluding zero, technology intensity positively predicted health issues through Technostress. When school support was at a high level (M + 1SD), with a 95% CI = [−0.06, 0.14] consisting of zero, the indirect effect was insignificant. With the improvement of school support, the direct effect increased while the indirect effect decreased. These indicated that technology intensity positively predicted teachers’ health issues directly and indirectly with increased school support levels. Therefore, Hypothesis 4 was supported.

**Table 7:** Decomposition of direct and mediating effects.

Personal health	School support	Effect size	Boot SE	Boot LLCI	Boot ULCI	Ratio
Direct effect	−0.88(M-1SD)	0.15	0.06	0.01	0.04	53.38%
	0.12(M)	0.15	0.04	0.0003	0.07	61.86%
	1.12(M + 1SD)	0.14	0.06	0.02	0.02	78.86%
Indirect effect of technostress	−0.88(M-1SD)	0.13	0.03	0.08	0.19	46.62%
	0.12(M)	0.09	0.03	0.04	0.14	38.14%
	1.12(M + 1SD)	0.04	0.05	−0.06	0.14	21.14%

## 6 Discussion

Since the outbreak of COVID-19, teachers worldwide have increasingly relied on technology to facilitate the transition from traditional classroom settings to frequent remote education. Primary school teachers, who cater to younger students, may face greater challenges in managing student affairs and maintaining engagement than middle school, high school, or university teachers. As a result, primary school teachers might be more susceptible to Technostress, a contemporary issue stemming from technology use that can lead to emotional and physical problems for users. With appropriate intervention and policies, primary school teachers may experience Technostress, contributing to burnout and, ultimately, threatening student well-being and overall educational sustainability.

Through the lens of COR, this study is among the first to focus exclusively on primary school teachers’ Technostress, offering valuable insights for policymakers and school administrators. In particular, this research contributes to the existing literature by elucidating the complex yet clear relationships among primary school teachers’ Technostress, technology use intensity, school support, work–family conflicts, and personal health issues, significantly impacting teacher performance and student outcomes.

Firstly, primary school teachers engage with technology intensively for approximately 4 to 6 h daily during weekdays, serving various purposes such as lesson preparation, teaching, administrative duties, and communication with students’ parents. Due to this intense interaction with technology, teachers generally report moderate to high levels of Technostress. This finding is not surprising, considering that teaching is widely recognized as one of the most stressful professions globally (Golembiewski et al., 1983), and teachers rank among the top occupations frequently acquiring emerging technological knowledge. This observation also clarifies why, among the five stressors of Technostress, techno-uncertainty and techno-overload received the highest ratings. After all, emerging technologies are rapidly evolving worldwide, and when primary school teachers use technology for 4 to 6 h daily, they inevitably face the risk of overload. Moreover, when teachers are not instructing, they must utilize technology to address multifaceted purposes by switching between tasks rather than resolving issues sequentially. Such sporadic technology use compels teachers to multitask, likely contributing to increasing Technostress (Mark et al., 2008; Ragu-Nathan et al., 2008; Tarafdar et al., 2015).

Secondly, there were notable demographic variations in primary school teachers’ Technostress. For instance, it was shown that male primary school teachers felt greater Technostress than female teachers. This is consistent with Ragu-Nathan et al. (2008) study on corporate employees but conflicts with Ong and Lai's (2006) or Shah et al.’s findings. A possible explanation could be the imbalanced gender distribution in primary schools, where male teachers may be perceived as more tech-savvy and receive more technology-related tasks, as males generally feel more confident using technology than females (Yau and Cheng, 2012). Consistently, a study on gender differences in EFL teachers’ use of technology also reported that female teachers used technology less than their male counterparts in teaching (Mahdi and Al-Dera, 2013).

Regarding age, it was found that older primary school teachers experienced higher Technostress levels. This result contradicts Ragu-Nathan et al. (2008) finding that older employees might feel less Technostress than their younger counterparts. Such a disagreement could result from occupational differences. Consistent with societal expectations, teachers often hold higher moral standards for their jobs than other professions, such as salespersons or technical workers (Siu and Lam, 2009). In other words, although older teachers may be less proficient in learning emerging technologies, they remain as motivated as younger teachers in fulfilling professional duties and maintaining a positive reputation, particularly as they approach retirement.

Additionally, Technostress levels increase as primary school teachers’ teaching years accumulate. This finding aligns with the age variable results. It could be explained that the longer teachers stay in the profession, the more technologies they need to master alongside other job-related duties and tasks, resulting in heightened Technostress. Furthermore, there was a statistically significant difference between headteachers’ and non-headteachers’ Technostress. This may be explained by the fact that headteachers are often expected to fulfill more diverse roles than non-headteachers, such as establishing culture identification, managing resources, and maintaining a good relationship with parents (Argyriou and Iordanidis, 2014), and thus likely to suffer from greater work-related stress than regular teachers (Scott et al., 2021).

The demographic differences in Technostress suggest that school administrators should pay particular attention to senior female headteachers. While allocating technology-related tasks based on demographic variances may be unfair or unreasonable, providing additional psychological and technical support for the groups of teachers with greater needs is appropriate and acceptable. This support can help them build positive beliefs and attitudes toward technology use, strongly indicating their eventual use and efficiency (Russell et al., 2003). Providing targeted training and professional development opportunities for these teachers can improve their technical proficiency and reduce their Technostress. This might include offering workshops, mentoring programs, or technology coaching tailored to the specific needs of senior female headteachers or other at-risk groups. Also, fostering a supportive school culture where teachers feel comfortable discussing their Technostress and seeking help from colleagues or administrators can be beneficial. In summary, understanding the demographic differences in Technostress among primary school teachers is crucial for school administrators to create targeted support and intervention programs. Schools can create a more inclusive and supportive environment by addressing senior female headteachers and other at-risk groups’ unique needs, ultimately enhancing teacher performance and student outcomes.

Thirdly, our study demonstrated a direct association between primary school teachers’ Technostress levels, work–family conflicts, and health issues. In other words, higher levels of Technostress correlated with more frequent occurrences of work–family conflicts and health issues. This finding partially supports Tarafdar et al. (2007) claim that Technostress would indirectly impact technology users’ work-life conflict. The inconsistency may be attributed to the significant difference in technology integration levels between now and 2007, when smartphones and social networking began gaining popularity. As reported by primary school teachers in our study, they spend at least 4 to 6 h daily using technology for teaching-related tasks, occupying a substantial portion of their work and personal time.

Consequently, it is reasonable to speculate that the intensive use of technology directly, rather than indirectly, results in work–family conflicts. Furthermore, our study found that Technostress directly influences health issues among primary school teachers, such as visual fatigue, headaches, and sore shoulders. The higher the Technostress, the more frequent these symptoms arise. This aligns with previous findings that Technostress can lead to physical problems, including fatigue (Salanova et al., 2014) and insomnia (Porter and Kakabadse, 2006). To address these issues, we recommend schools invest in facilities or hire fitness professionals to help teachers alleviate fatigue and physical discomfort. For example, Latino et al. (2021) conducted a study on 40 teachers, finding that an eight-week yoga intervention resulted in statistically significant improvements in teachers’ bodily and emotional awareness and prevention of professional burnout.

Fourthly, our study indicates that primary school teachers’ technology intensity directly impacts their work–family conflicts and personal health and indirectly affects them through Technostress. These dual paths suggest that reducing technology intensity is one way to alleviate work–family conflicts and health issues for primary school teachers while addressing their Technostress is another viable approach. Notably, the indirect impact is more pronounced than the direct impact when primary school teachers receive higher levels of school support. In other words, teachers are more likely to experience work–family conflicts and health issues due to technology-induced psychological stress rather than prolonged hours of technology use. This finding contradicts Kyriacou and Chien (2004) conclusion that primary teachers considered the most effective stress-reduction strategy to be a simple decrease in workload. The discrepancy may stem from the nearly two-decade gap between the two studies or differences in research methods. Our conclusion is based on statistical analysis, while Kyriacou and Chien’s restated teachers’ opinions.

Once again, our results underscore the importance of supporting teachers during technological reforms through adequate measures that alleviate their perceived stress from technology use. For example, the announcement of administrative tasks could be confined to a fixed time frame rather than distributed randomly throughout the day. This change would allow teachers to focus more on teaching-related tasks and reduce the burden of multitasking. Moreover, schools must help teachers understand the explicit requirements of educational policies related to technology use (Kyriacou and Chien, 2004) so that they feel less pressured and better supported by such policies.

Lastly, our study found that school support moderates the indirect relationship between technology intensity, work–family conflicts, and health issues. As school support increases, the indirect impact of technology intensity on teachers’ work–family conflicts and personal health becomes less apparent through the agency effect of Technostress. In other words, enhancing school support will likely reduce primary teachers’ Technostress, a finding consistent with most previous studies’ hypotheses (Wang et al., 2008; Salanova et al., 2014).

Our findings suggest that when primary school teachers have low technology intensity, perceived school support does not affect their Technostress. However, when the intensity level is high, greater perceived school support corresponds to less Technostress. Therefore, school leaders should provide technical support that facilitates effective learning and teachers’ use of emerging technologies. Additionally, schools should offer timely comprehensive training to bridge the gap between teachers’ willingness to integrate technology and their full capacity to integrate it (Liu, 2007). Moreover, Gaudioso et al. (2017) considered the lack of coping mechanisms a cause of Technostress; thus, schools should invite psychology experts to share knowledge about coping with upcoming or existing stress. In summary, our study highlights the importance of school support in mitigating the negative impacts of technology intensity on primary school teachers’ work–family conflicts and health issues.

### 6.1 Implications

Given the above, the findings of this study highlight the significance of addressing Technostress among primary school teachers to enhance their well-being and support a conducive learning environment. Drawing upon the COR theory, several practical strategies can be employed by relevant stakeholders to mitigate Technostress and promote teacher well-being.

To address the Technostress experienced by teachers, school administrators, and education board officials should prioritize comprehensive technological training and support programs. These initiatives will empower teachers with the necessary skills to navigate technology integration effectively, contributing to a sense of resource gain rather than loss. Tailored training, focusing on individual teachers’ needs and challenges, will ensure that they build and maintain valuable resources (knowledge and skills) to cope with the demands of technology use.

Furthermore, fostering a supportive school culture, recognizing teachers’ efforts, and promoting open communication can act as resources to buffer against Technostress. By providing social support and a sense of belonging, teachers can perceive an increase in resource availability, thereby reducing the impact of Technostress on their well-being. Peer support networks and mentorship programs can be valuable additions to create an environment of collaboration and resource exchange among teachers.

Considering the observed relationship between Technostress and work–family conflicts, it is essential to implement work-life balance initiatives. Reducing administrative burden and offering flexible scheduling options can replenish teachers’ resources outside of their professional lives, mitigating the negative effects of Technostress on their personal well-being.

Moreover, acknowledging age and gender differences in Technostress experiences is crucial, especially for senior female headteachers who may be more susceptible to Technostress. Inclusive decision-making processes involving teachers of diverse backgrounds will ensure that technological policies consider varying needs. This approach gives teachers control over their resources, promoting a positive work environment.

Regular well-being assessments will aid stakeholders in monitoring teachers’ Technostress levels and identifying areas of concern. These assessments will enable targeted interventions, such as resource enrichment programs and continuous professional development, to address specific stressors effectively.

Lastly, technology providers can reduce Technostress by collaborating with schools, gathering feedback from teachers, and promptly addressing usability issues. By involving teachers in product development and offering responsive customer support, technology providers enhance teachers’ resource gain from using technology.

By implementing these measures collectively and accounting for the nuanced interplay of variables, such as age, gender, and seniority, school communities can empower teachers, minimize Technostress, and optimize the educational experience for teachers and students, leading to a healthier and more productive teaching and learning environment. Through the COR theory lens, these informed practices promote resource preservation and enhancement for primary school teachers, ultimately fostering a positive and supportive school culture.

### 6.2 Limitations

Firstly, the present study solely employed survey research, which is quantitative in nature, and the findings could have been further strengthened by integrating qualitative methods such as conducting in-depth interviews. Specifically, representative groups of teachers (such as those differentiated by gender and age) could have been interviewed to gain a more comprehensive understanding of the sources or triggers of Technostress. Secondly, it is important to consider that the participants were recruited via convenience sampling from two provinces in eastern China. Therefore, the results may not be generalizable to populations that differ significantly from those in our study. Thirdly, the participants were predominantly female teachers, accounting for approximately 80.3% of the total sample, while male teachers constituted only 19.7%. This noticeable gender imbalance could potentially impact the generalizability of our findings, especially concerning variations in Technostress experiences specific to different genders. However, our primary objective was not to directly compare the experiences of male and female teachers but rather to provide a comprehensive understanding of Technostress in the teaching profession. Fourthly, the variable “school support” was measured solely through self-reported subjective questions, and objective criteria were not used in its assessment. To further investigate the multifaceted effects of school support, future researchers could increase the number of related questions and expand its assessment criteria to include subjective and objective evaluations. Lastly, the Technostress level was measured after the Pandemic, so it is difficult to empirically conclude that teachers’ Technostress increased solely due to the Pandemic. To gain a more nuanced understanding of the underlying causes of Technostress, future research could conduct longitudinal studies and measure teachers’ Technostress at multiple time points under different circumstances.

Overall, it is important to acknowledge these limitations and for future researchers to address them to further contribute to the field of Technostress and its impact on primary school teachers.

## 7 Conclusion

Since the advent of the COVID-19 pandemic, teachers worldwide have faced a significant upsurge in Technostress due to the extensive use of online teaching and related technologies. This study, conducted through the lens of the Conservation of Resources (COR) theory, aimed to investigate primary school teachers’ Technostress and its relationship with critical factors such as perceived school support, technology intensity, work–family conflicts, and personal health.

The findings of this study revealed that primary school teachers in China have been extensively utilizing technologies daily, resulting in a moderate to high level of Technostress. Notably, there were statistically significant differences based on gender, age, teaching years, and headteacher duties, underscoring the need for targeted support for senior female headteachers who were found to be more susceptible to Technostress.

The study further identified a positive and significant correlation between primary teachers’ Technostress, work–family conflicts, and technology-induced health issues. Moreover, technology intensity was found to directly impact primary teachers’ work–family conflicts and personal health, while school support played a moderating role. School support was observed to diminish the indirect impact of technology intensity on work–family conflicts and health issues, emphasizing the importance of a supportive school environment.

To mitigate the negative effects of technology use on teachers’ well-being and teaching performance, policymakers and school administrators should prioritize measures to reduce the duration of technology use and ensure that teachers are regularly updated on emerging technologies. Special attention should be given to senior female headteachers, who may require tailored support to cope with the challenges of technology integration.

Additionally, providing psychological consultation services and opportunities for physical exercise can enable primary teachers to effectively manage Technostress, minimize work–family conflicts, and enhance their personal well-being and physical health. By addressing these critical issues, this study underscores the need for targeted interventions and support mechanisms to promote teachers’ well-being and teaching performance. Enhancing teacher preparation and professional development in the context of technology use will ultimately foster the sustainability of education for students and their families.

In conclusion, this study contributes to the ongoing discourse on technology integration in education and highlights the importance of understanding and addressing Technostress among primary school teachers. Through the insights gained from the COR theory, stakeholders can implement evidence-based practices that empower teachers, mitigate Technostress, and support the well-being of teachers in the changing landscape of education. These efforts are crucial in ensuring the quality and sustainability of education for the benefit of students and teachers alike.

## Data availability statement

The dataset supporting the conclusions of this article will be made available by the authors, upon reasonable request.

## Ethics statement

The studies involving humans were approved by Qingdao University Teacher’s College Academic Committee. The studies were conducted in accordance with the local legislation and institutional requirements. The participants provided their written informed consent to participate in this study. Written informed consent was obtained from the individual(s) for the publication of any potentially identifiable images or data included in this article.

## Author contributions

ZW: Writing – original draft. LZ: Supervision, Writing – review & editing. XW: Data curation, Methodology, Writing – original draft. LL: Formal analysis, Writing – original draft. CL: Conceptualization, Resources, Writing – review & editing.
